# Supplementary material for: Morphology of travel routes and the organization of cities
Source: Nat Commun. 2017 Dec 20;8:2229. doi: 10.1038/s41467-017-02374-7 (PMC5738436; doi:10.1038/s41467-017-02374-7)
Supplement: Supplementary file 1 — Supplementary information [file 41467_2017_2374_MOESM1_ESM.pdf]

# Morphology of travel routes and the organization of cities Supplementary Information

Minjin Lee, Hugo S. Barbosa, Hyejin Youn, Petter Holme, and Gourab Ghoshal

## Supplementary Note 1
## Materials and Methods

### Collecting data from API service

Origin-Destination (OD) pairs were generated having the cities' centers as the reference points. In the absence of a worldwide definition of a city center, we used the coordinates collected and provided by the 'latlong.net'service, for each of the 92 cities. Having such coordinates as reference center points, we systematically computed *theoretical* OD pairs corresponding to combinations of discretized radial and angular ranges.

In many cases, the theoretical points have no access to the streets networks, being therefore approximated to their closest point within the networks. Such approximation is done automatically by the OpenStreetMap (OSM) routing API. However, to avoid the large discrepancies between a requested (theoretical) point and those returned by the OSM API, we applied two post-processing filters. First, if the distance of a returned OD coordinate is off by more than 1km from the center, we exclude such routes from the data. Second, we also excluded those routes whose lengths are longer than $3s + 1km$, where $s$ is the geodesic distance between the origin and destination points.

### Data description

For each of the 92 cities, the maximum total number of unique driving routes is 630. However, after filtering out those discrepant routes and OD pairs, for each of the radii values, the average number of valid routes we analyzed were 575.9 (2km), 532.8 (5km), 461.7 (10km), 391.0 (15km), 349.3 (20km) and 254.7 (30km). Supplementary Table 1 also describes the detour index (DI) (i.e., the ratio $d/r$ between the travel distance $d$ and geodesic distance $r$) for fastest (F) and shortest (S). Avg. waypoint represents the average number of route points as returned by the OSM API for each city.

Supplementary Table 1: **Data description**

| | | | | Avg. Number of Valid routes for each radius | | | | | |
|---|---|---|---|---|---|---|---|---|---|
| City | DI (S) | DI (F) | Avg. waypoint | 2km | 5km | 10km | 15km | 20km | 30km |
| Abidjan | 1.576 | 1.659 | 188.88 | 584 | 589 | 571 | 386 | 328 | 177 |
| Accra | 1.497 | 1.595 | 158.81 | 587 | 611 | 421 | 266 | 216 | 169 |
| Ahmadabad | 1.392 | 1.491 | 233.76 | 625 | 612 | 610 | 481 | 539 | 378 |
| Ankara | 1.48 | 1.599 | 367.45 | 612 | 619 | 580 | 571 | 420 | 397 |
| Atlanta | 1.307 | 1.478 | 386.32 | 599 | 621 | 624 | 629 | 630 | 630 |
| Baghdad | 1.6 | 1.766 | 212.38 | 550 | 602 | 606 | 496 | 339 | 220 |

| | | | | | | | | | |
|---|---|---|---|---|---|---|---|---|---|
| Bandung | 1.573 | 1.657 | 380.07 | 619 | 580 | 536 | 418 | 211 | 309 |
| Bangalore | 1.338 | 1.506 | 354.03 | 616 | 623 | 627 | 628 | 630 | 628 |
| Bangkok | 1.505 | 1.634 | 275.5 | 476 | 608 | 597 | 606 | 606 | 485 |
| Barcelona | 1.504 | 1.68 | 384.15 | 597 | 342 | 266 | 195 | 203 | 182 |
| Belo Horizonte | 1.525 | 1.682 | 456.71 | 612 | 617 | 619 | 606 | 470 | 516 |
| Berlin | 1.297 | 1.47 | 405.61 | 629 | 626 | 622 | 622 | 619 | 626 |
| Bogota | 1.566 | 1.661 | 455.55 | 608 | 621 | 606 | 583 | 583 | 427 |
| Boston | 1.374 | 1.519 | 626.85 | 576 | 616 | 571 | 520 | 495 | 405 |
| Buenos Aires | 1.353 | 1.511 | 178.25 | 613 | 520 | 259 | 252 | 231 | 209 |
| Cairo | 1.5 | 1.614 | 241.77 | 607 | 600 | 601 | 597 | 558 | 481 |
| Cape town | 1.471 | 1.562 | 275.8 | 479 | 357 | 251 | 320 | 187 | 66 |
| Chennai | 1.409 | 1.566 | 163.64 | 613 | 624 | 270 | 228 | 210 | 167 |
| Chicago | 1.387 | 1.551 | 206.56 | 590 | 346 | 228 | 210 | 210 | 190 |
| Chongqing | 1.72 | 1.769 | 287.77 | 448 | 521 | 578 | 480 | 403 | 304 |
| Dalian | 1.486 | 1.535 | 129.62 | 615 | 533 | 210 | 133 | 100 | 65 |
| Dar es salaam | 1.488 | 1.54 | 169.06 | 609 | 595 | 590 | 516 | 217 | 141 |
| Delhi | 1.409 | 1.483 | 262.27 | 618 | 604 | 608 | 617 | 621 | 453 |
| Dhaka | 1.568 | 1.648 | 244.44 | 519 | 593 | 462 | 489 | 380 | 293 |
| Dongguan | 1.569 | 1.61 | 159.03 | 587 | 500 | 512 | 425 | 542 | 516 |
| Dubai | 1.629 | 1.656 | 154.63 | 570 | 443 | 394 | 240 | 212 | 182 |
| Fuzhou | 1.656 | 1.719 | 481.95 | 608 | 586 | 503 | 492 | 469 | 426 |
| Guadalajara | 1.431 | 1.623 | 277.36 | 613 | 630 | 623 | 469 | 522 | 219 |
| Guangzhou | 1.55 | 1.644 | 312.96 | 512 | 558 | 594 | 556 | 537 | 502 |
| Hangzhou | 1.473 | 1.574 | 167.2 | 613 | 616 | 587 | 544 | 569 | 416 |
| Hanoi | 1.453 | 1.556 | 218.4 | 622 | 606 | 612 | 570 | 571 | 439 |
| Harbin | 1.593 | 1.638 | 119.13 | 543 | 456 | 406 | 243 | 425 | 178 |
| Ho Chi Minh City | 1.438 | 1.534 | 257.03 | 594 | 628 | 623 | 580 | 579 | 502 |
| Houston | 1.298 | 1.458 | 308.5 | 595 | 627 | 626 | 630 | 628 | 626 |
| Hyderabad | 1.38 | 1.533 | 346.95 | 623 | 620 | 622 | 621 | 622 | 546 |
| Istanbul | 1.462 | 1.615 | 335.32 | 454 | 569 | 431 | 452 | 315 | 187 |
| Jakarta | 1.461 | 1.618 | 236.13 | 604 | 620 | 620 | 551 | 348 | 246 |
| Johannesburg | 1.394 | 1.575 | 226.01 | 589 | 623 | 608 | 619 | 592 | 567 |
| Kabul | 1.656 | 1.678 | 219.18 | 351 | 508 | 376 | 179 | 161 | 27 |
| Khartoum | 1.538 | 1.615 | 228.41 | 603 | 606 | 339 | 357 | 405 | 217 |
| Kinshasa | 1.579 | 1.648 | 213.51 | 600 | 604 | 586 | 465 | 209 | 86 |
| Kolkata | 1.396 | 1.523 | 322.11 | 630 | 608 | 617 | 447 | 336 | 145 |
| Kuala Lumpur | 1.549 | 1.648 | 337.27 | 564 | 595 | 575 | 533 | 433 | 393 |
| Lagos | 1.633 | 1.726 | 283.73 | 592 | 442 | 400 | 465 | 240 | 66 |
| Lahore | 1.533 | 1.627 | 147.2 | 584 | 602 | 437 | 365 | 340 | 136 |
| Lima | 1.434 | 1.574 | 241.57 | 614 | 618 | 545 | 281 | 232 | 61 |
| London | 1.295 | 1.488 | 619.26 | 615 | 628 | 629 | 623 | 625 | 627 |
| Los Angeles | 1.294 | 1.463 | 329.49 | 614 | 624 | 616 | 629 | 630 | 427 |
| Luanda | 1.518 | 1.699 | 138.07 | 616 | 623 | 589 | 251 | 182 | 100 |
| Madrid | 1.416 | 1.557 | 414.53 | 609 | 607 | 600 | 583 | 486 | 521 |
| Manila | 1.399 | 1.501 | 271.95 | 623 | 586 | 481 | 390 | 296 | 294 |
| Medan | 1.478 | 1.565 | 161.89 | 625 | 620 | 591 | 561 | 335 | 43 |
| Mexico City | 1.411 | 1.608 | 314.42 | 611 | 608 | 624 | 612 | 580 | 455 |
| Miami | 1.486 | 1.589 | 168.23 | 546 | 567 | 296 | 171 | 153 | 135 |
| Milan | 1.348 | 1.519 | 562.1 | 621 | 611 | 624 | 628 | 627 | 630 |

| | | | | | | | | | |
|---|---|---|---|---|---|---|---|---|---|
| Monterrey | 1.413 | 1.571 | 218.96 | 623 | 622 | 589 | 487 | 324 | 245 |
| Moscow | 1.437 | 1.564 | 384.64 | 600 | 616 | 618 | 619 | 586 | 581 |
| Mumbai | 1.536 | 1.654 | 235.4 | 588 | 615 | 417 | 283 | 238 | 183 |
| Nagoya | 1.21 | 1.397 | 423.62 | 629 | 629 | 630 | 627 | 629 | 595 |
| Nairobi | 1.541 | 1.669 | 273.97 | 603 | 487 | 590 | 511 | 458 | 426 |
| Nanjing | 1.473 | 1.576 | 186.61 | 609 | 546 | 565 | 505 | 475 | 351 |
| New York | 1.405 | 1.561 | 338.79 | 536 | 615 | 605 | 624 | 559 | 429 |
| Osaka | 1.279 | 1.471 | 426.54 | 620 | 626 | 623 | 494 | 463 | 494 |
| Paris | 1.283 | 1.465 | 536.62 | 619 | 624 | 628 | 627 | 629 | 630 |
| Philadelphia | 1.331 | 1.505 | 411.91 | 623 | 598 | 617 | 620 | 622 | 629 |
| Phoenix | 1.328 | 1.454 | 318.56 | 619 | 625 | 629 | 622 | 592 | 489 |
| Pune | 1.461 | 1.536 | 397.43 | 602 | 616 | 618 | 511 | 615 | 373 |
| Qingdao | 1.527 | 1.592 | 125.23 | 617 | 400 | 174 | 191 | 170 | 180 |
| Quanzhou | 1.684 | 1.717 | 148.72 | 168 | 201 | 432 | 390 | 239 | 173 |
| Rio de Janeiro | 1.674 | 1.758 | 337.41 | 555 | 476 | 401 | 307 | 225 | 247 |
| Rome | 1.455 | 1.593 | 491.23 | 602 | 613 | 598 | 617 | 617 | 400 |
| San Francisco | 1.457 | 1.565 | 367.15 | 611 | 579 | 344 | 265 | 227 | 228 |
| Sao Paulo | 1.405 | 1.565 | 490.48 | 603 | 618 | 628 | 625 | 621 | 573 |
| Shanghai | 1.351 | 1.496 | 215.91 | 567 | 623 | 625 | 616 | 558 | 366 |
| Shenyang | 1.414 | 1.53 | 125.36 | 615 | 620 | 611 | 563 | 507 | 371 |
| Shenzhen | 1.569 | 1.655 | 344.57 | 571 | 545 | 595 | 556 | 526 | 369 |
| Singapore | 1.518 | 1.599 | 152.25 | 524 | 525 | 361 | 295 | 223 | 190 |
| Surabaya | 1.574 | 1.661 | 232.05 | 573 | 579 | 324 | 325 | 285 | 185 |
| Surat | 1.441 | 1.522 | 152.92 | 609 | 563 | 400 | 458 | 340 | 187 |
| Suzhou | 1.459 | 1.544 | 140.48 | 598 | 602 | 549 | 593 | 542 | 416 |
| Sydney | 1.475 | 1.58 | 316.83 | 588 | 611 | 526 | 292 | 222 | 148 |
| Taipei | 1.537 | 1.672 | 540.47 | 597 | 599 | 608 | 523 | 597 | 360 |
| Tehran | 1.505 | 1.649 | 295.42 | 622 | 603 | 603 | 571 | 417 | 300 |
| Tianjin | 1.467 | 1.568 | 191.99 | 600 | 604 | 586 | 465 | 209 | 86 |
| Tokyo | 1.229 | 1.487 | 448.21 | 630 | 629 | 630 | 623 | 593 | 495 |
| Toronto | 1.304 | 1.414 | 229.89 | 624 | 593 | 226 | 190 | 189 | 171 |
| Washington DC | 1.312 | 1.494 | 566.67 | 624 | 606 | 627 | 615 | 624 | 618 |
| Wuhan | 1.558 | 1.646 | 211.51 | 519 | 570 | 566 | 523 | 536 | 403 |
| Xiamen | 1.57 | 1.626 | 176.6 | 589 | 482 | 375 | 347 | 305 | 265 |
| Xian | 1.423 | 1.506 | 171.11 | 625 | 607 | 611 | 591 | 516 | 562 |
| Yangon | 1.469 | 1.513 | 162.23 | 623 | 624 | 595 | 514 | 340 | 176 |
| Zhengzhou | 1.454 | 1.532 | 133.43 | 613 | 590 | 607 | 533 | 493 | 353 |

## Routes as street samples

If we consider only the subset of the streets within the 30km radius of our analyses, on average, the shortest and fastest routes covered approximately 24% and 18 % of the overall streets networks, respectively (Supplementary Figure 2a). However, when we talk about different routing approaches, faster arterial roads and minor residential streets are going to respond for different aspects of the route optimization, and therefore they are expected to cover different samples of a road network. The distribution of road types being sampled by each routing method is depicted in the Supplementary Figure 2 b&c. As explained in the main text, arterial roads such as motorway and trunk roads are much more relevant for fastest routes than for shortest routes, reflecting in how frequently they appear in each type of route. Supplementary Table 2 shows the fraction of the overall streets networks being sampled by the shortest and fastest

routes for each city as well as the ratio between the two fractions. Supplementary Figure 3 & 4 depict the participation of the most frequent road types for each city.

Supplementary Table 2: **Sample fraction of routes**

| City | Shortest route | Fastest route | Fastest route/Shortest route |
|---|---|---|---|
| Abidjan | 0.149 | 0.117 | 0.786 |
| Accra | 0.247 | 0.179 | 0.722 |
| Ahmadabad | 0.285 | 0.226 | 0.791 |
| Ankara | 0.288 | 0.191 | 0.662 |
| Atlanta | 0.183 | 0.141 | 0.768 |
| Baghdad | 0.282 | 0.183 | 0.651 |
| Bandung | 0.188 | 0.165 | 0.874 |
| Bangalore | 0.240 | 0.151 | 0.627 |
| Bangkok | 0.154 | 0.124 | 0.808 |
| Barcelona | 0.195 | 0.125 | 0.641 |
| Berlin | 0.186 | 0.133 | 0.713 |
| Bogota | 0.193 | 0.125 | 0.649 |
| Boston | 0.248 | 0.163 | 0.659 |
| Buenos Aires | 0.278 | 0.192 | 0.688 |
| Cairo | 0.139 | 0.103 | 0.738 |
| Cape Town | 0.163 | 0.117 | 0.718 |
| Chennai | 0.177 | 0.112 | 0.632 |
| Chicago | 0.209 | 0.126 | 0.605 |
| Chongqing | 0.507 | 0.486 | 0.959 |
| Dalian | 0.540 | 0.511 | 0.947 |
| Dar es Salaam | 0.101 | 0.083 | 0.821 |
| Delhi | 0.092 | 0.061 | 0.662 |
| Dhaka | 0.185 | 0.133 | 0.717 |
| Dongguan | 0.181 | 0.184 | 1.013 |
| Dubai | 0.126 | 0.100 | 0.796 |
| Fuzhou | 0.367 | 0.364 | 0.991 |
| Guadalajara | 0.278 | 0.154 | 0.554 |
| Guangzhou | 0.271 | 0.248 | 0.915 |
| Hangzhou | 0.343 | 0.330 | 0.963 |
| Hanoi | 0.354 | 0.278 | 0.784 |
| Harbin | 0.408 | 0.374 | 0.915 |
| Ho Chi Minh City | 0.238 | 0.188 | 0.791 |
| Houston | 0.213 | 0.129 | 0.605 |
| Hyderabad | 0.097 | 0.056 | 0.579 |
| Istanbul | 0.204 | 0.105 | 0.516 |
| Jakarta | 0.178 | 0.125 | 0.700 |
| Johannesburg-East Rand | 0.222 | 0.143 | 0.643 |
| Kabul | 0.154 | 0.118 | 0.767 |
| Khartoum | 0.176 | 0.112 | 0.639 |
| Kinshasa | 0.135 | 0.095 | 0.705 |
| Kolkata | 0.197 | 0.129 | 0.656 |
| Kuala Lumpur | 0.170 | 0.118 | 0.697 |
| Lagos | 0.309 | 0.231 | 0.747 |
| Lahore | 0.181 | 0.136 | 0.754 |

| | | | |
|---|---|---|---|
| Lima | 0.231 | 0.143 | 0.620 |
| London | 0.214 | 0.129 | 0.602 |
| Los Angeles | 0.250 | 0.146 | 0.582 |
| Luanda | 0.264 | 0.177 | 0.671 |
| Madrid | 0.203 | 0.136 | 0.669 |
| Manila | 0.158 | 0.108 | 0.682 |
| Medan | 0.321 | 0.231 | 0.721 |
| Mexico City | 0.222 | 0.140 | 0.630 |
| Miami | 0.215 | 0.145 | 0.676 |
| Milan | 0.226 | 0.131 | 0.580 |
| Monterrey | 0.236 | 0.136 | 0.576 |
| Moscow | 0.117 | 0.083 | 0.706 |
| Mumbai | 0.208 | 0.167 | 0.802 |
| Nagoya | 0.172 | 0.052 | 0.305 |
| Nairobi | 0.275 | 0.221 | 0.802 |
| Nanjing | 0.298 | 0.276 | 0.927 |
| New York | 0.244 | 0.155 | 0.634 |
| Osaka | 0.153 | 0.066 | 0.435 |
| Paris | 0.221 | 0.116 | 0.527 |
| Philadelphia | 0.297 | 0.189 | 0.636 |
| Phoenix | 0.165 | 0.099 | 0.596 |
| Pune | 0.368 | 0.277 | 0.754 |
| Qingdao | 0.404 | 0.414 | 1.026 |
| Quanzhou | 0.406 | 0.414 | 1.019 |
| Rio de Janeiro | 0.201 | 0.150 | 0.745 |
| Rome | 0.289 | 0.206 | 0.713 |
| San Francisco | 0.213 | 0.135 | 0.633 |
| Santiago | 0.354 | 0.275 | 0.778 |
| Sao Paulo | 0.260 | 0.150 | 0.575 |
| Shanghai | 0.265 | 0.210 | 0.792 |
| Shenyang | 0.430 | 0.391 | 0.910 |
| Shenzhen | 0.165 | 0.146 | 0.885 |
| Singapore | 0.222 | 0.178 | 0.802 |
| Surabaya | 0.197 | 0.155 | 0.785 |
| Surat | 0.459 | 0.391 | 0.852 |
| Suzhou | 0.264 | 0.259 | 0.982 |
| Sydney | 0.208 | 0.149 | 0.715 |
| Taipei | 0.230 | 0.164 | 0.712 |
| Tehran | 0.238 | 0.148 | 0.624 |
| Tianjin | 0.439 | 0.397 | 0.903 |
| Tokyo | 0.170 | 0.058 | 0.341 |
| Toronto | 0.190 | 0.109 | 0.577 |
| Washington DC | 0.179 | 0.115 | 0.645 |
| Wuhan | 0.415 | 0.359 | 0.866 |
| Xiamen | 0.342 | 0.337 | 0.986 |
| Xian | 0.406 | 0.355 | 0.874 |
| Yangon | 0.136 | 0.094 | 0.695 |

## Supplementary Note 2
## Comparison with network centrality measures

In this section, we compare the spatial distribution of inness with different centrality measures of relevance in the context of transport networks. The objective here is to verify to what extent inness can deliver additional relevant information for which traditional measures of centrality do not account.

Without loss of generality, one can say that the inness of a certain point in the road network of a city is the result of the aggregation of the characteristics of the routes that potentially pass through that point. Indeed, it captures geometric aspects of the network since it is a measure based on the curvature of the roads along a route. It also reflects the structure of the network since it is a metric influenced by topology and network connectivity. Moreover, it also reflects the long-range topographical and geometric relationships since it is not a local measure of centrality but is actually a property of the route. Thus, if a certain point participates in routes with both positive and negative inness, and of equal magnitude, that point is expected to exhibit a null inness. On the other hand, if the inness of the routes crossing a given point is predominantly positive (or negative), we can safely say that that particular point is part of a functional sub-structure of the network with specific geometric characteristics.

We compared the inness with network centrality measures capable of reflecting different facets of the structures, such as topology, locality and geometry. The centrality measures of choice were:

**Closeness** is a measure of centrality that determines how close a particular node in the network is to all other nodes. Clearly, in the context of spatial networks in a finite area, the nodes with the greatest closeness are those close to the centroid of the network. In the context of spatial networks, closeness is essentially a geometric metric as the actual topology of the network has no importance in determining the centrality of a node.

**Eccentricity** - the eccentricity of a given node is the largest geodesic distance between that node and any other node in the network. Contrasting with the closeness centrality, eccentricity, by definition, does not reflect the geometric characteristics of the network being mostly a topological metric.

**Degree** centrality is a measure of local connectivity of a node in which the importance of a node is determined by the number of nodes directly connected to it. It is primarily a measure of the local connectivity of the nodes, having no long-range correlations.

**Betweenness** is a measure that ascribes importance to those nodes lying along the shortest paths between other pairs of nodes such that the more the shortest paths crossing a node, the higher its betweenness centrality will be. It is probably one of the most investigated centrality measures, especially in the context of road networks.

In Supplementary Figure 1 the inness profile, along with the four centrality measures for three large European cities. In each panel, values above the average are colored in red, and below that, in blue. The first striking feature we can observe is that the inness profile is very distinct from the ones produced by the centrality measures. For instance, none of the centrality measures managed to capture the concentric circumferential patterns produced by the ring-like structures in the network, manifested as low inness zones.

Another interesting pattern we can observe is the region of extreme inness profiles, suggesting the presence of large *detour* spots such that routes traveling through those regions have no option but to drive inward. For example, a closer look at the East of London, where routes across the River Thames are being pushed away (low inness in blue) or pulled towards the city center (high inness in red), is a direct consequence of the absence of bridges in that area. Such functional insights from the systems are not possible from the observation of the standard centrality measures in isolation.

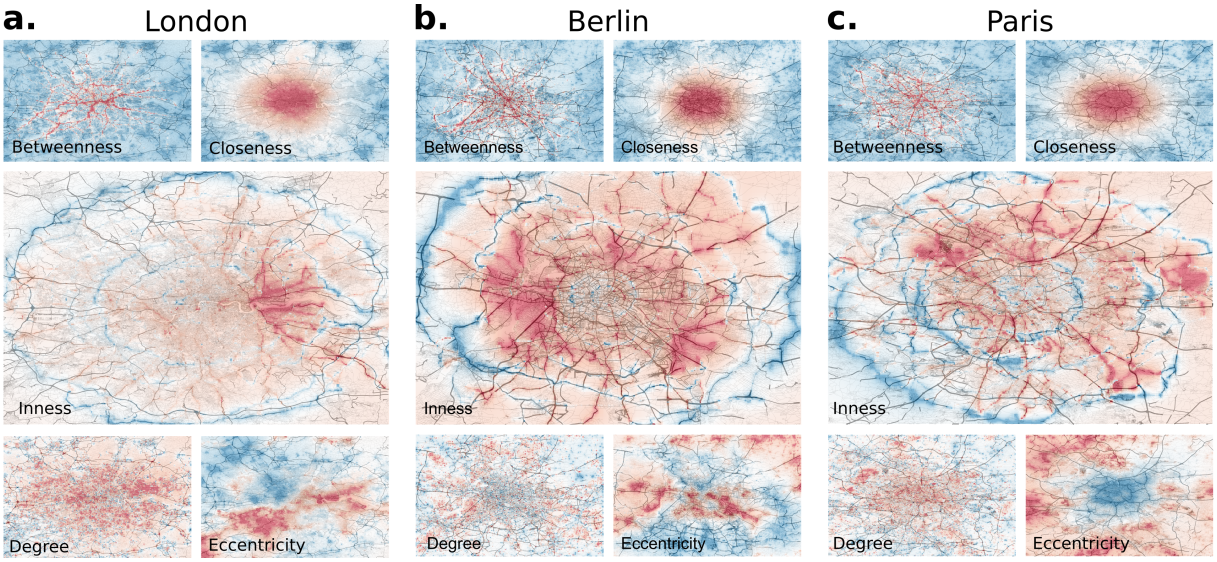


Supplementary Figure 1: **Comparison of inness and network metrics** Spatial distribution of inness in comparison with different network centrality measures. Here we show spatial profile of the inness (center panel) compared with the spatial profiles of different centrality measures (bottom and top panels) for three different cities, **a.** London, **b.** Berlin and **c.** Paris.

# Supplementary Note 3
# Types of roads

We summarize the statistics of road types for our sample cities. We also compare the routes samples with the complete road network within the same boundary we used for the inness calculation. The complete road network data was collected from the OpenStreetMap repository using a service[1]. In Supplementary Figure 2 we show the distribution of the road types in our routes data for each. See `http://wiki.openstreetmap.org/wiki/Key:highway#Values` for detailed information about the road type labels and their meanings.

[1] `https://extract.bbbike.org/`

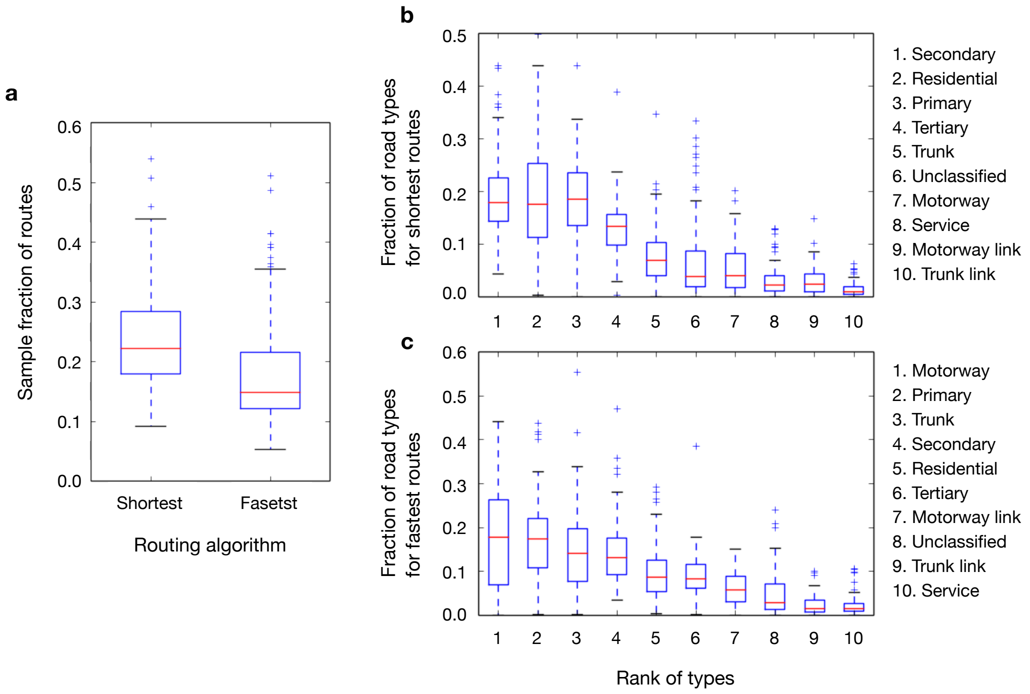


Supplementary Figure 2: **Summary of route information for 92 cities A** Sample fraction of routes among entire street networks in each urban area for 92 cities described as boxplots (both shortest and fastest routes). **B&C** Fraction of road types for sampled routes. **B** shows the shortest routes and **C** represents the fastest routes.

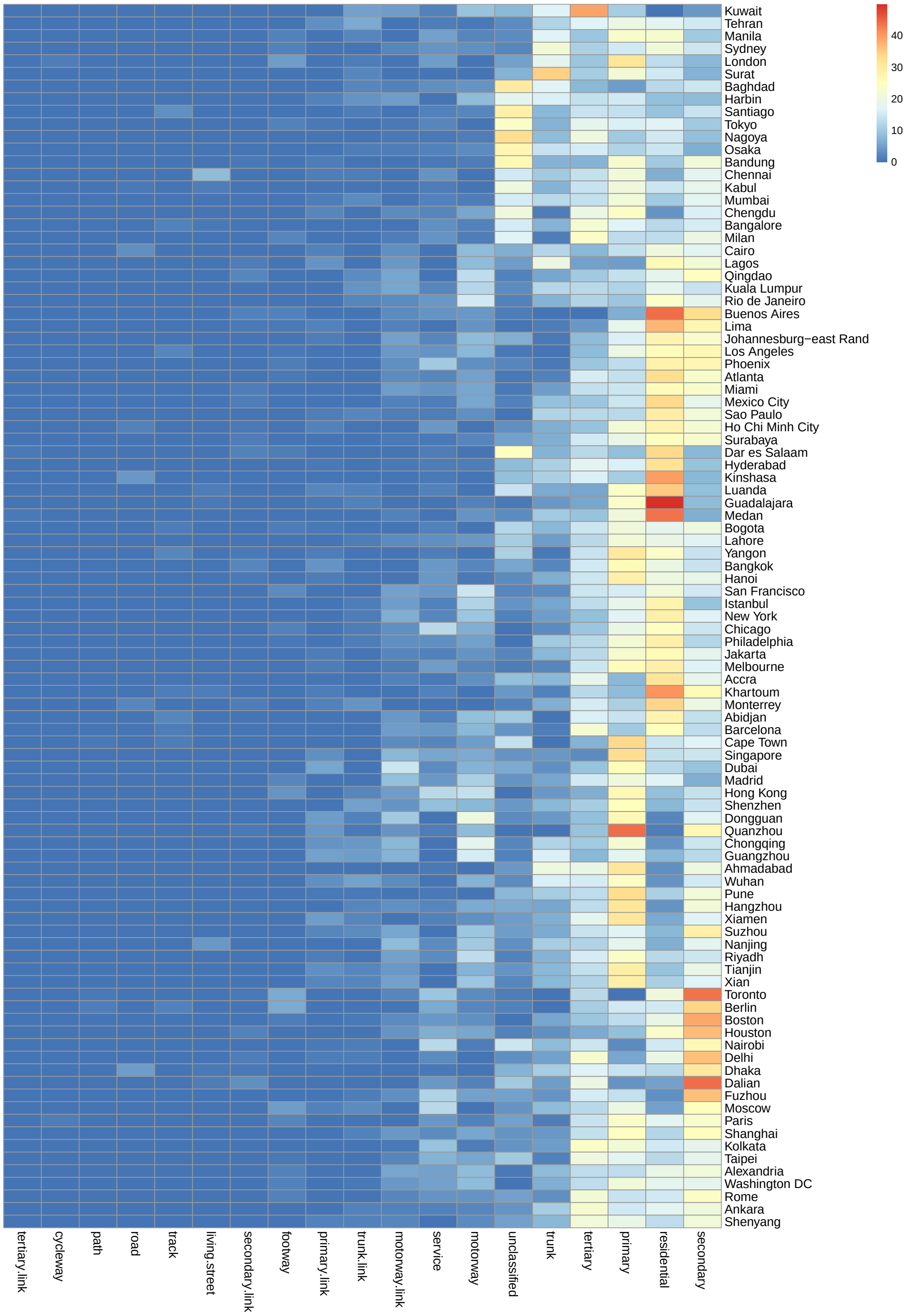


Supplementary Figure 3: **Fraction of road types for shortest routes**

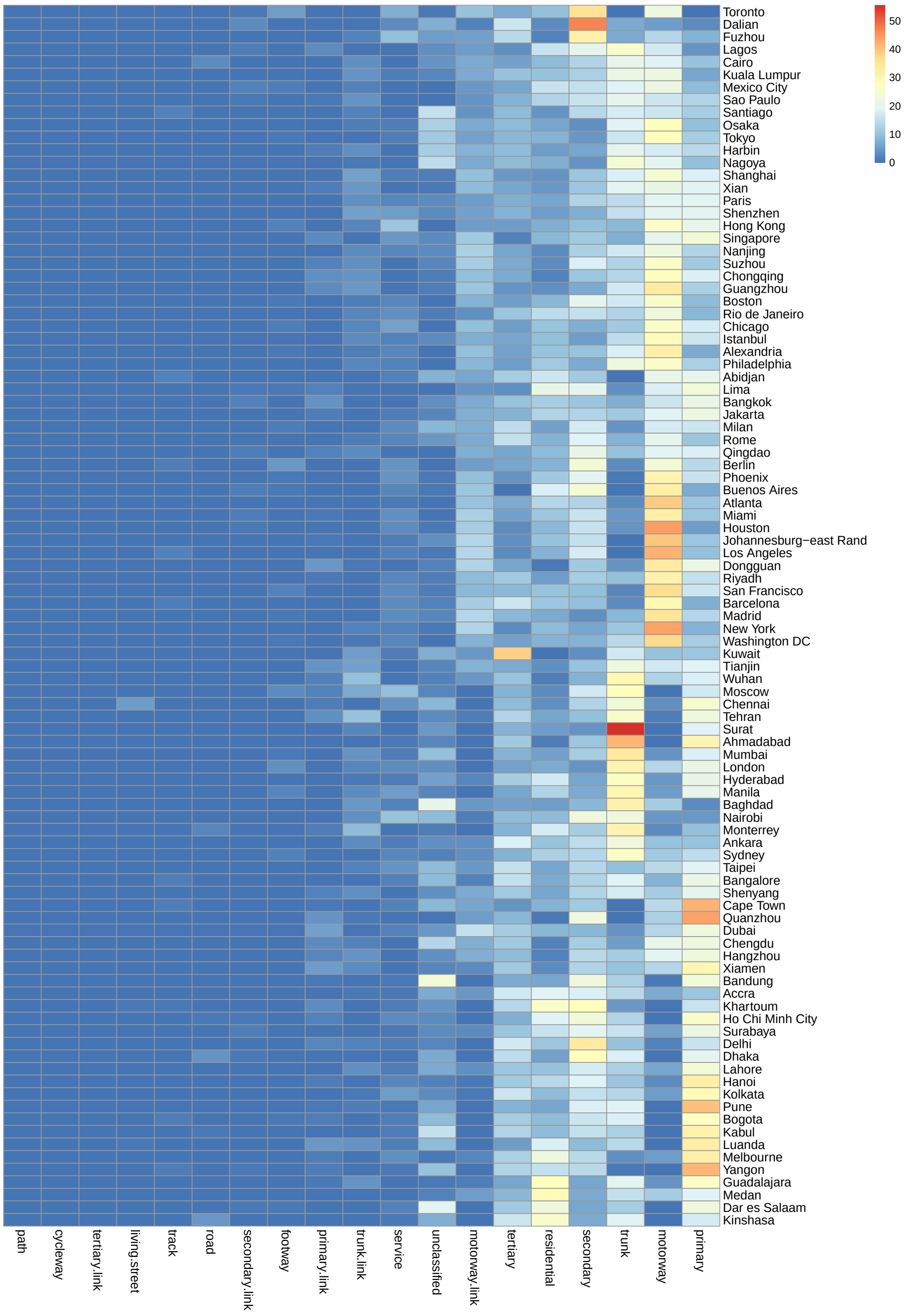


Supplementary Figure 4: **Fraction of road types for fastest routes**

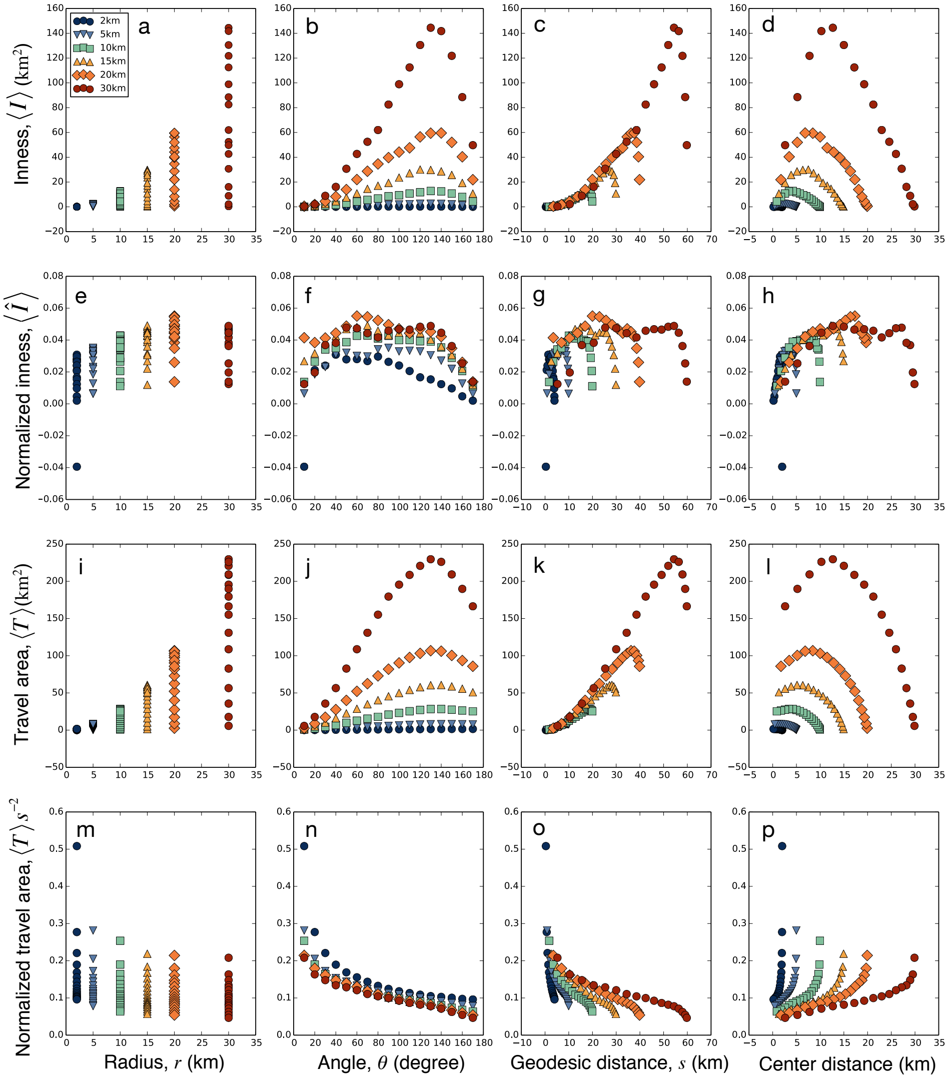


Supplementary Figure 5: **Summary statistics for inness and travel area**

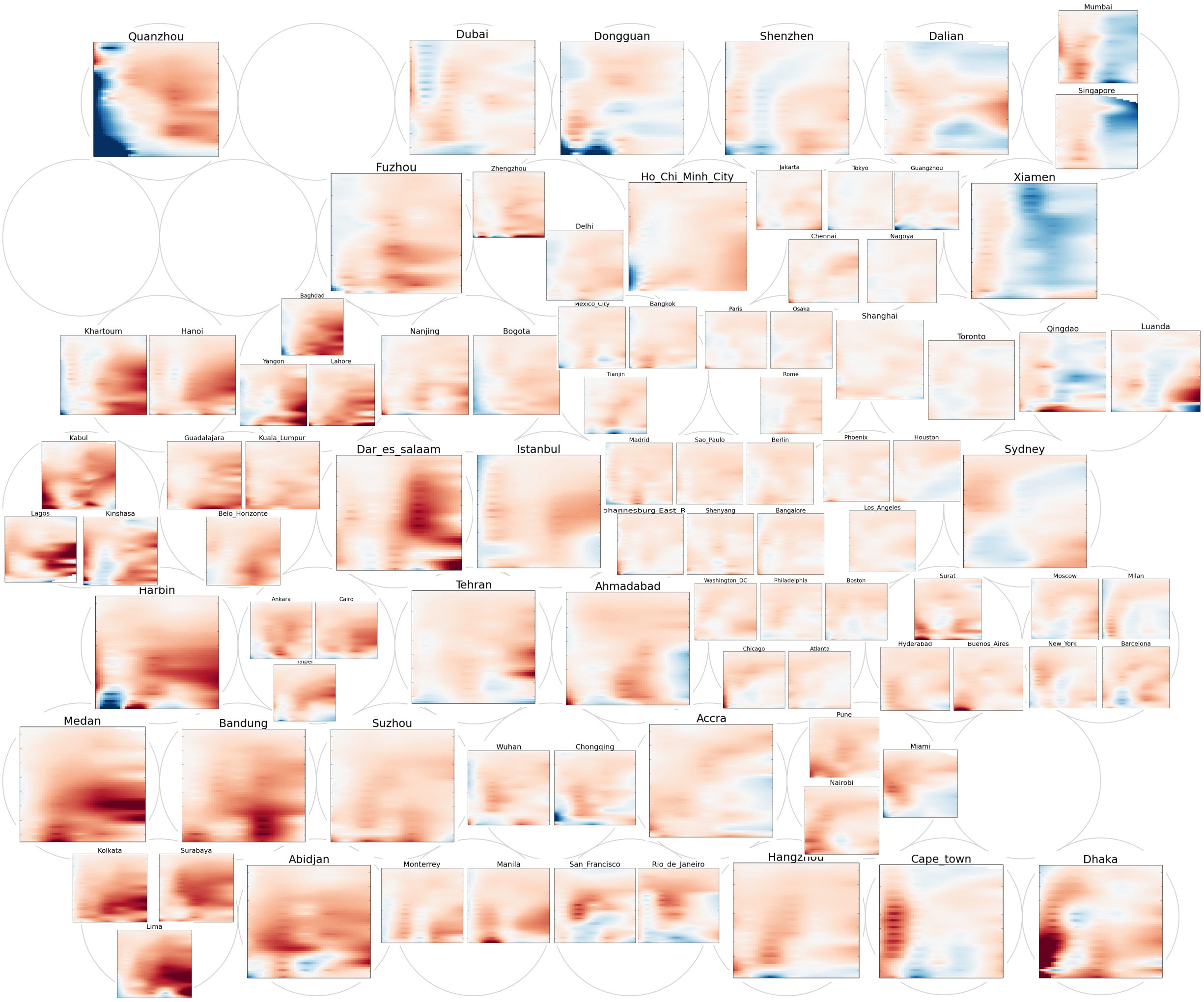


Supplementary Figure 6: **Normalized inness of shortest routes for 89 cities** The normalized inness patterns of shortest routes for individual cities are arranged by its similarity. The cities with similar inness patterns are close to each other and the cities at the both ends are most different to each other. The values range from -0.3 (blue) to 0.3 (red). The cities were clustered using a Self-Organizing Map (SOM) to assign positions in a 2D plane.

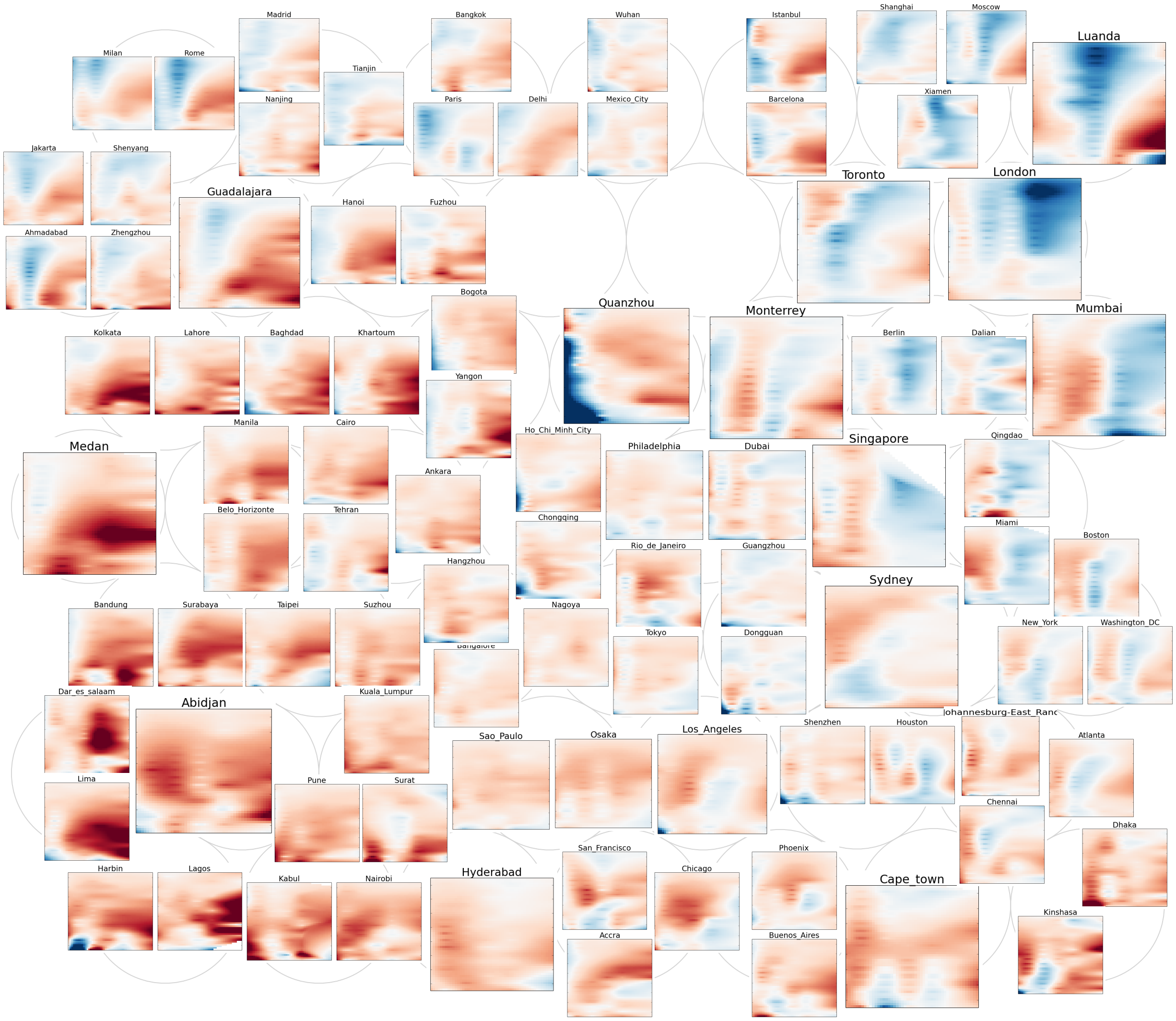


Supplementary Figure 7: **Normalized inness of fastest routes for 89 cities** Method and scale same as in Supplementary Figure 6

# Supplementary Note 4
# Metric for road structure

We suggest three metrics to measure various facet of infrastructural and geographical features. We use same road networks data used and explained in Supplementary Note 3

**Road length** is the total length of motorways, trunks, secondary, primary and tertiary roads for each city.

**Level of geographical constraints (GC)** represents the overall fraction of the city that is not covered by the road network due to the presence of *barriers*. Here, we define a barrier as an area of the city that is unaccessible via public roads, being either natural (e.g., forests and mountains) or artificial (e.g., a large industrial or military site). To calculate the GC we generated 10,000 uniformly distributed points within the same area of our study and computed its relative distance to the nearest point of the road network. More precisely, GC can be defined as

$$GC = rd/rc,$$

where $rd$ is the distance of the point to the closest street segment and $rc$ is the distance to the city center from the random point. For instance, if many random points in a particular area are closer to the center than to a road, GC becomes bigger, suggesting, therefore, the presence of a large barrier close to the center. In the Supplementary Figure 8a, the point $A$ is inside the urban area and have road segments nearby while the point $B$ is located in a mountainous area. Although the two points have similar $rc$ (the blue dashed line), $B$ has higher $rd$ (the red dashed line) than $A$ and consequently the GC of $B$ is higher than that of $A$. The term $rc$ accounts for barriers near the city center having a greater impact to routes than barriers of similar area in the periphery. In Supplementary Figure 9 we show two examples of representative cities with very different GC profiles.

As one can see, London has almost no regions of poor connectivity caused by geographical constraints. Mumbai, on the other hand, has many regions of little to no connectivity due to the presence of geographical constraints such as the large Sanjay Gandhi National Park (the brighter spot in the north-northeast region), and the Thane Creek, the inlet that isolates the city of Mumbai from the Indian mainland.

**Peripheral connectivity** represents the average value of all the acute angles of the *higher-level* peripheral roads, or more precisely, the motorways and trunks beyond a minimum distance from the center, in this case, 10km. These parameter choices are motivated by the reasonable assumption that a road segment with a high angle (or close to 90 degrees) is likely to be part of ring-like structure, which is presumably used more for connecting peripheries than spoke-like roads. The greater the average angle, the more likely it is that the peripheries are connected, thus acting as a proxy for the presence of circumferential roads. To calculate the angle between the center and a road segment, we draw the shortest line between the center and the middle point of a road segment and measure the angle between the line and the road segment, as depicted in Supplementary Figure 8b.

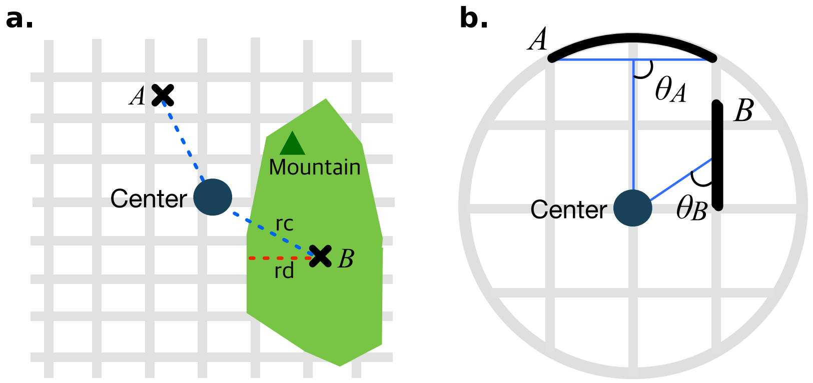


Supplementary Figure 8: **Schematic diagram of road structure metrics a** Geographical constraints (GC) **b** Peripheral connectivity (PC)

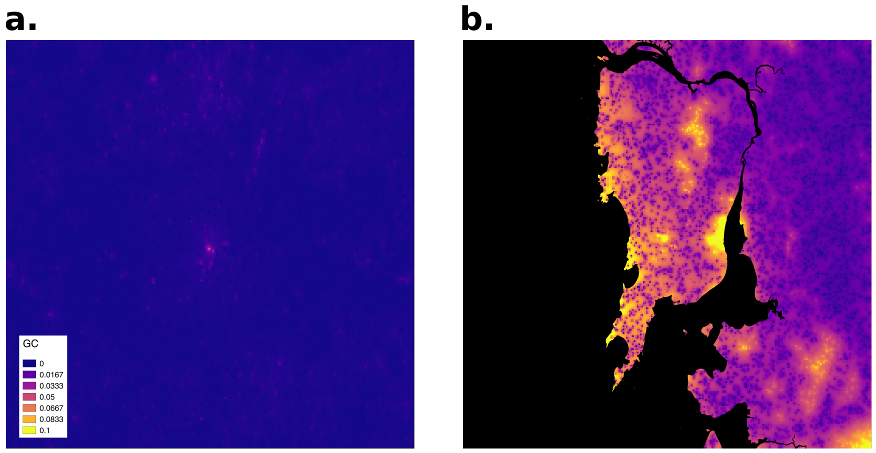


Supplementary Figure 9: **Examples of the spatial distribution of the geographical constraints (GC)** The values of geographical constraints (GC) for 10,000 random points are spatially mapped on two sample cities; **a** London and **b** Mumbai. The same color scheme is applied to both cities with a range from 0 to 0.1. Note that London is one of the cities with low average and standard deviation inness (i.e., LL group) whereas Mumbai is a low average and high standard deviation inness city (i.e., LH group).

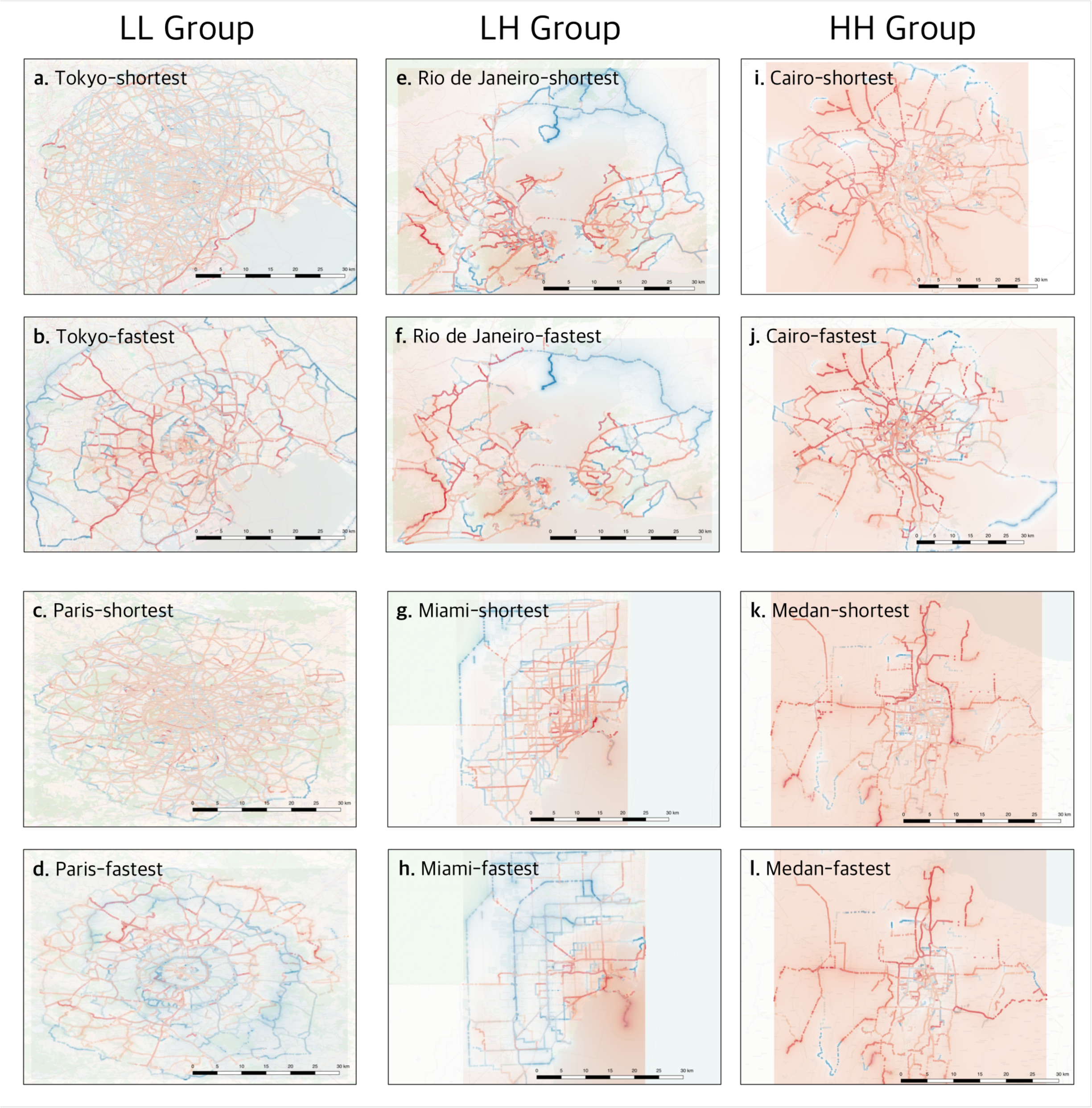


Supplementary Figure 10: **Spatial distribution of cities for each group.** Examples of cities of the types discussed in Fig. 4 of the main manuscript. The group LL, LH and HH are classified according to the standard deviation and average values of the inness (LL Group: Low standard deviation and low average (close to zero); LH Group: high standard deviation and low average; HH Group: High standard deviation and high average).

## Supplementary Note 5
## Outlier cities

Some cities such as Quanzhou, Dongguan, Qingdao, Kinshasa, Harbin, Surat and Kabul exhibit extremely high standard deviation in comparison with other cities (See SI, Section Fig for details on the outliers). For Quanzhou, Dongguan and Qingdao which have relatively low average inness, most part of these cities are shaped by geographical constraints such as the closeness to the coast or being along the path of a river. Just like the geographical constraints influence the shape of the routes of cities in the third category, similar barriers strongly affect these *outlier* cities. For instance, Kinshasa, Harbin, Surat and Kabul basically belong to the second category, i.e., a "hub and spoke" structure with strong positive inness signal. However these cities also have negative inness values due to lack of infrastructure (e.g., bridges) connecting different parts of the city across the rivers.

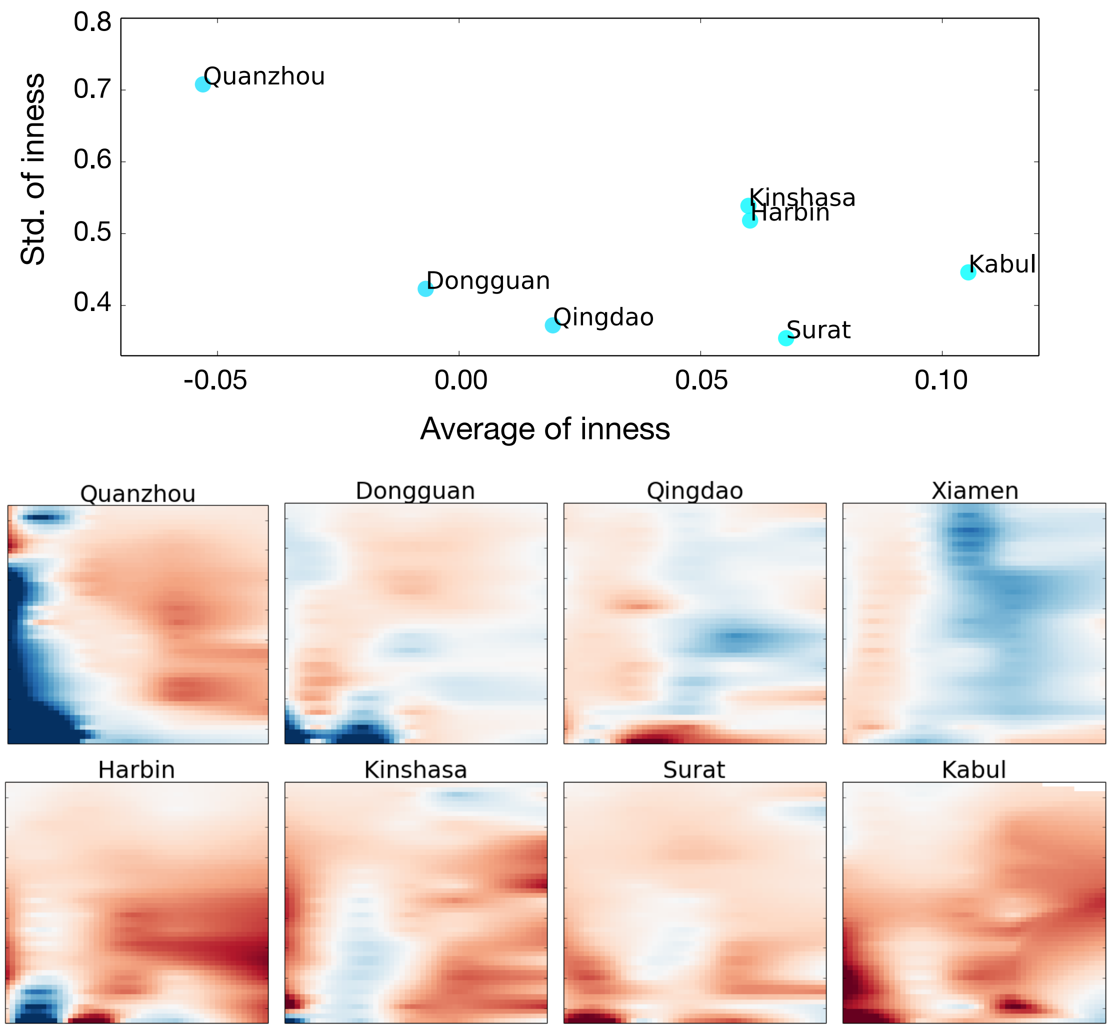


Supplementary Figure 11: **Outlier cities** Inness profiles of cities that do not fall into any of our categories. The values range from -0.3 (blue) to 0.3 (red)

# Supplementary Note 6
# The correlation between shortest and fastest routes

When we take into account the inness of the fastest routes, we are indirectly incorporating the influence of second-order structural features of the network such as roadway capacity and speed limits. The analysis of the faster routes, therefore, offers an additional perspective and to a certain point closer to the real operation of that structure.

However, a decidedly more elaborate picture about the structure of the road network can be obtained by means of a quantitative characterization of the geometric similarities and, above all, of the discrepancies between the shorter and faster routes.

This is because it is only through this comparison that we can verify where and with what magnitude the influence of the path capacity in the geometry of the routes occurs. For example, if for a given pair of origin and destination the shortest and fastest route have discrete inness profiles, this difference is only possible because the segments along the faster route are potentially more temporally efficient.

The correlation between the inness of the shortest and the fastest routes is a measure capable of revealing this difference between the two route types. In fact, those urban systems where the shortest and the fastest routes have little difference are those where any increases in distance are not offset by gains in terms of travel time. From a purely structural perspective this could be said to be an efficient road network such that the fastest routes are also the shorter routes.

Our hypothesis, however, is that it can occur for two main reasons: (1) due to greater homogeneity in terms of road capacity and/or (2) due to low road network capillarity. Therefore, we used three different correlation measures to classify cities according to their similarity profiles between the shortest and fastest routes.

## Classifying cities based on their Inness profiles

Although we are not claiming that the cities can be *naturally* classified into different discrete groups, here we show that the correlation between the inness of shortest ($I_s$) and fastest ($I_f$) routes can be used as a metric to classify cities. Thus, we computed three correlation measures, namely Pearson correlation, Spearman's rank order correlation and Kendall rank correlation. The measures were computed comparing the inness of the average shortest and fastest routes for each radius/angle value.

For each of the $\mathcal{N} \leq 36$ routes with radius $r$ and angular distance $\theta$ we computed the average $I_S$ and $I_F$, with the inequality being due to the existence of unfeasible paths for certain OD pairs, and measured the correlation coefficients between the two inness arrays. The rationale to use three correlation metrics is that this way we can characterize the said dissimilarities in a higher dimensionality space, accounting not only for the absolute values of the inness but also for the ranks of the $(r, \theta)$ pairs in terms of their inness .

We then applied a hierarchical clustering method to produce *a* partitioning of the cities based on their similarities in terms of their fastest and shortest inness profiles. The method is a standard complete linkage clustering method in which the maximum possible distance between points belonging to different groups is sought. Supplementary Figure 12 shows the dendrogram of the partitioning.

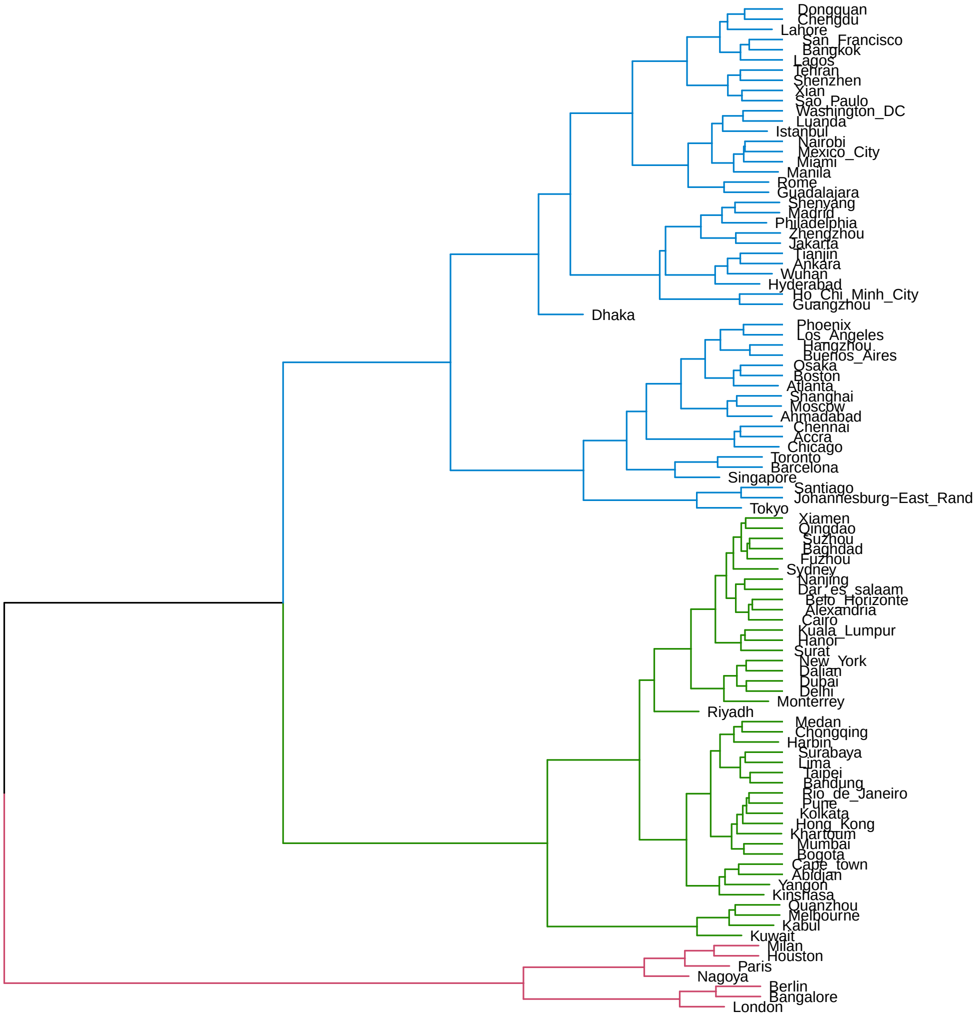


Supplementary Figure 12: **Hierarchical clustering of cities based on three correlation measures** The colors illustrate a 3-clusters partitioning.

Next we computed the within-clusters sum of squared deviations (WCSS) to quantify how much of the variance could be explained by partitioning the cities into$k$ clusters. Clearly, a perfect partitioning would be one in which each cluster contains one single city. Supplementary Figure 13 shows the WCSS as we increase the number of clusters. As we can see, most of the variance can be explained by three clusters and only very little variance is explained by increasing the number of clusters from 4 to 5, suggesting that the best partition would be one with $k = 3$ or $k = 4$. Bellow we show one partitioning obtained from the Pearson correlation coefficient $\rho$.

**Type I** ( $\rho \leq 0.22$) London, Bangalore, Berlin, Paris, Nagoya, Atlanta, Shenyang, Milan, Shanghai, Tokyo, Houston;

**Type II** ( $0.22 < \rho \leq 0.6$) Philadelphia, Ahmadabad, Zhengzhou, Chennai, Buenos Aires, Madrid, Phoenix, Johannesburg Barcelona, Guangzhou, Boston, Miami, Toronto, Moscow, Los Angeles, Hyderabad, Guadalajara, Chicago, Tianjin, Hangzhou, Ankara, Wuhan, Osaka, Washington DC, Accra, Ho Chi Minh City, Rome, Jakarta, Kuala Lumpur, Xian;

**Type III** ($0.6 < \rho \leq 1$) Shenzhen, Dubai, Manila, Surat, Luanda, Tehran, Pune, Kolkata, Dhaka, Nairobi, Mexico City, São Paulo, Lahore, Bangkok, Hanoi, Xiamen, Qingdao, Baghdad, Suzhou, Dalian, Nanjing, Monterrey, Fuzhou, Istanbul, Delhi, New York, Dongguan, Sydney, Cairo, Lima, Abidjan, Yangon, Chongqing, Khartoum, Bandung, Surabaya, Harbin, Mumbai, Bogota, Taipei, Rio de Janeiro, Kabul, Kinshasa, Quanzhou.

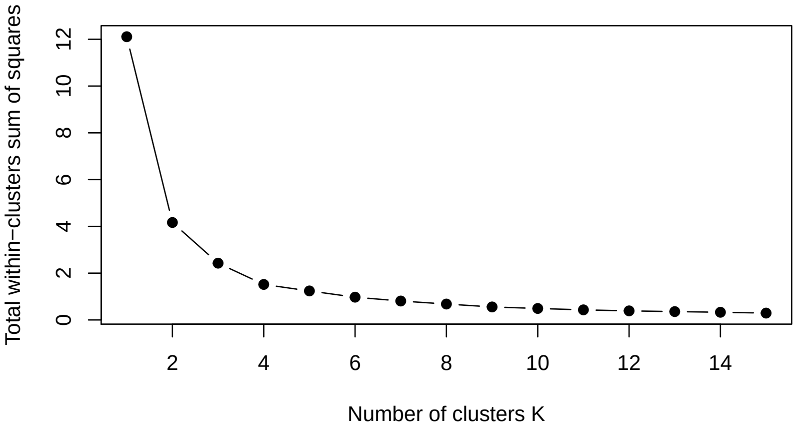


Supplementary Figure 13: **Within-clusters sum of squared deviations (WCSS) as a function of the number of clusters** $k$ Most of the variance can be explained by three clusters and only very little variance is explained by increasing the number of clusters from 4 to 5, suggesting that the best partition would be one with $k = 3$ or $k = 4$.

## Socio-economic indicators

As we presented in the main manuscript, the (dis)similarities between the inness profiles produced by the shortest and fastest routes are often rooted on the level of development of the road infrastructure, which in turn is driven by the socio-economic development of the cities. We then explored the correlation between the $I_s$ and $I_f$ with three relevant indicators that could reflect the said stages of developments, namely the *productivity index (PI)*, the *infrastructure development index (IDI)* and the *GDP per capita* of the cities. The first two indexes (PI and IDI) are part of the *City Prosperity Index*, to date, the most comprehensive measure of the development of a city, developed by the United Nations program for human settlements (UN-Habitat). Each one of the six CPI indexes (including the PI and IDI) is defined in terms of an array of other indicators such as household income, economic specialization and housing infrastructure. The decision to employ the PI and IDI is motivated by the fact that these are the indexes more closely related to the structural development of the cities than other ones. For more details on the CPI indexes we refer the interested reader to the UN-Habitat Methodology and Metadata report [2].

The third indicator we used, i.e., the GDP per-capita of the cities, is based on the GDP@Risk estimate, a projected GDP of the cities based on the World City Risk Index — a risk-assessment metric developed by the Cambridge Centre for Risk Studies and published on the Lloyd's City Risk Index. More precisely, the index is a projection from 2015-2025 of the GDP accounting for different risk factors for the 301 world's major cities. More detailed information on the methodology can be found in the report 'World City Risk 2025: Part 2 Methodology' [3].

The decision to use a *projected* GDP – instead of the official estimated nominal GDPs officially published by the governments – is justified by three main reasons. The nominal GDP of a city is subject to some volatility due to many internal and external factors, contrasting with the transportation infrastructure of a city that tend to evolve over longer periods of time. Moreover, there are a lot of methodological variation in the way the nominal GDPs are estimated, especially for non-OECD cities. Additionally, the most recent data of the official GDPs does not necessarily correspond to the same period for different cities.

On the other hand, the projected GDP of the cities is a standardized metric based on the same scientific methodology for all the cities accounting for many factors of internal and external origin, from present infrastructure to potential natural disasters. Moreover, the GDP projection can reflect with a reasonable precision the *potentialities* of growth for a city, in which the level of development of the infrastructure plays a major role.

## New York

Contrasting with other large developed urban cities (type I), New York exhibited similar inness pattern between the shortest routes and fastest routes. The reason for such phenomena can be related to the geography of the motorways in the New York metropolitan area. Unlike other cities, New York does not have strong ring-like motorway structure in its periphery, which often are the preferred structures when it comes to congestion reduction and travel-times optimization. Instead, it has many radial and grid-like motorways, which has a limited effect on the inness patterns, as shown in the spatial distribution of fastest routes. Such particularity of the motorways of New York gives it unique inness characteristics, although further investigations regarding other factors (e.g., socioeconomic characteristics) is necessary.

[2] `http://cpi.unhabitat.org/sites/default/files/resources/CPI%20METADATA.2016.pdf`

[3] `http://cambridgeriskframework.com/wcr`

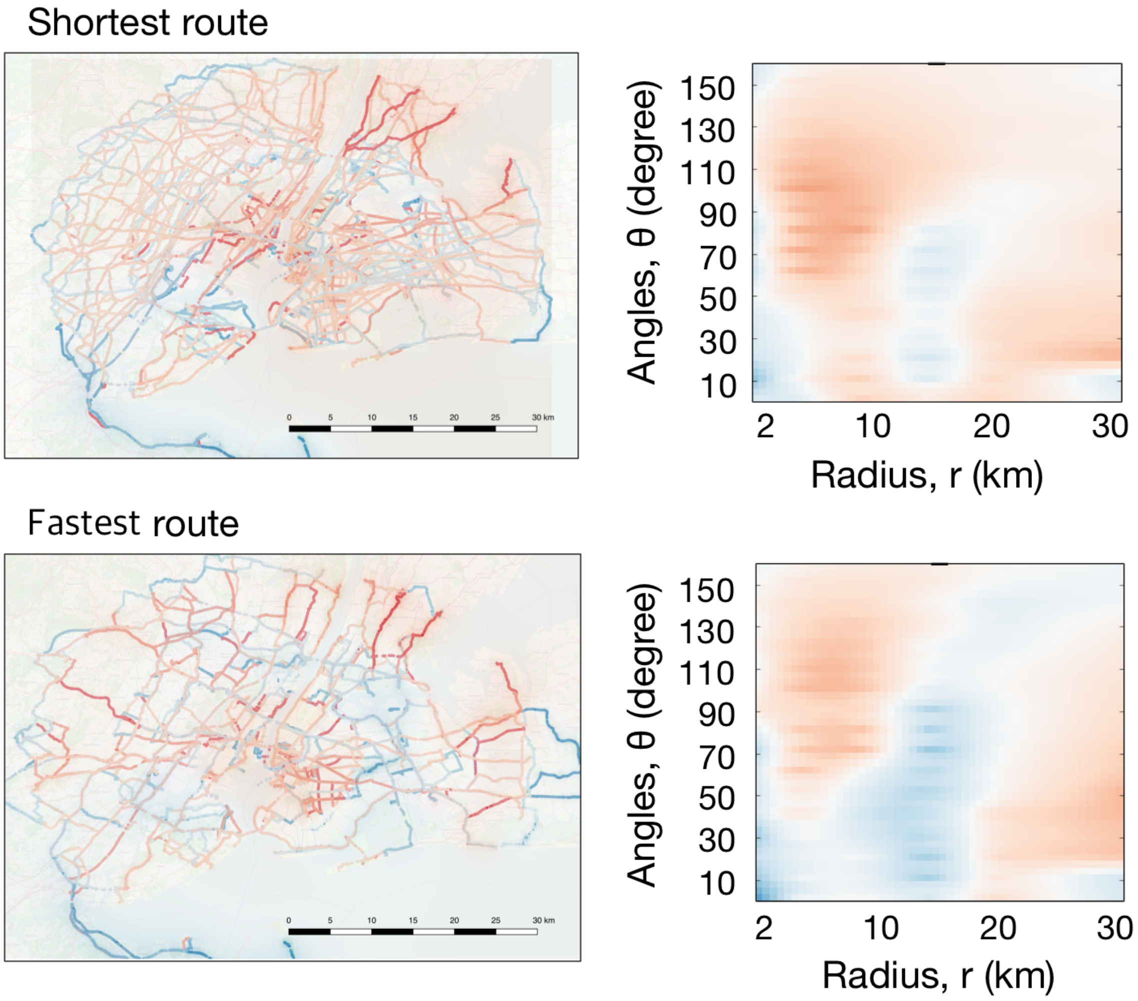


Supplementary Figure 14: **Inness pattern and spatial distribution of New York.**
